# 556. Ruxolitinib for the Management of Severe Pneumonia Caused by SARS-CoV-2. Exploring the Combination with dexamethasone

**DOI:** 10.1093/ofid/ofab466.754

**Published:** 2021-12-04

**Authors:** Aaron Molina, Alejandro Olmedo-Reneaum, Maria Del Rocio Reyes-Paniagua, Mariana Molina

**Affiliations:** 1 ISSSTE, Mexico City, Distrito Federal, Mexico; 2 IM resident @ Médica Sur, Mexico City, Distrito Federal, Mexico; 3 Instituto de Seguridad y Servicios Sociales de los Trabajadores del Estado, Ciudad de Mexico, Distrito Federal, Mexico; 4 McMaster University, Hamilton, Ontario, Canada

## Abstract

**Background:**

Mexico is one of the top five countries with a higher mortality rate of hospitalized patients of 30.1%. Since COVID-19 has been associated with immune dysregulation and hyper inflammation, JAK-12 inhibitors have been tested to reduce IL6 production. Studies have shown improvements when using ruxolitinib (rxb) in severely hospitalized patients with COVID-19. These have included patients in combination with corticosteroids such as dexamethasone (dxm). This work aims to test the response of hospitalized patients with severe or critical COVID-19 treated with rxb with or without dxm.

**Methods:**

An experimental, open, prospective study in a single third-level hospital in Mexico was performed. The primary outcome was favorable clinical response defined as withdrawal or decline of supplementary oxygen. Secondary outcomes such as mean hospital stay, improvement in systemic inflammatory response parameters, and mortality were also evaluated. Statistical differences for baseline and final measure and the use and not use of dxm were estimated. The study included adults with SARS-CoV-2 infection confirmed with polymerase chain reaction, radiological pneumonia, and oxygen saturation less than 90%. Rxb was administered 5mg/12hrs/15days, IV dxm 6mg/day/10days.

**Results:**

The final sample was 108 adults with complete information and informed consent. Sixty-two patients (57%) received only rxb. There were no differences between groups for any parameter at the beginning of treatment, and all patients were receiving supplemental oxygen. After 28-day follow-up, 70% reduce supplemental oxygen requirement (74% rxb and 71% rxb+dxm; p=0.628), 18% remained, and 2% increases support (1% with rxb, and 5% rxb+dxm; p< 0.001); 87% of patients were discharged (89% rxb and 85% rxb+dxm; p=0.603). In both groups, there was a significant reduction of CRP, LDH, and Ferritin on day 15. The mortality rate was 9% (no difference in groups; p=0.453), and a higher proportion died for *Pseudomonas aeruginosa* superinfection in the rxb+dxm group (p< 0.001).

Differences for support oxygen at baseline and discharge

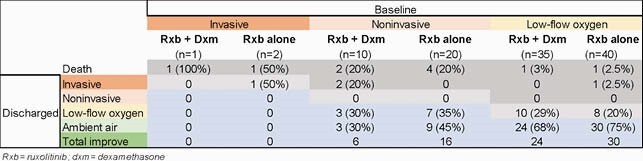

Final health outcomes of patients with severe or critical COVID-19 in a third-level hospital in Mexico

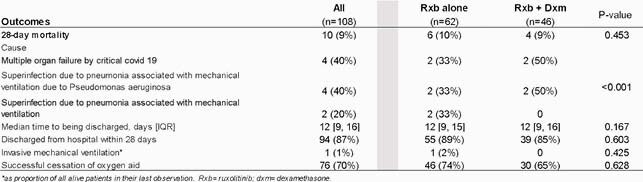

**Conclusion:**

The use of rxb could be considered as a treatment helping clinical improvement in hospitalized patients with severe COVID-19. Combination with dxm apparently did not add clinical benefits. It should be further evaluated.

**Disclosures:**

**All Authors**: No reported disclosures

